# Physiological mechanisms of drought-induced tree die-off in relation to carbon, hydraulic and respiratory stress in a drought-tolerant woody plant

**DOI:** 10.1038/s41598-017-03162-5

**Published:** 2017-06-07

**Authors:** Shin-Taro Saiki, Atsushi Ishida, Kenichi Yoshimura, Kenichi Yazaki

**Affiliations:** 1 Center for Ecological Research, Kyoto University, Otsu, Shiga 520-2113 Japan; 2 Faculty of Agriculture, Kyoto University, Kyoto, Kyoto, 606-8502 Japan; 3 Forestry and Forest Products Research Institute, Tsukuba, Ibaraki 305-8687 Japan

## Abstract

Drought-induced tree die-off related to climate change is occurring worldwide and affects the carbon stocks and biodiversity in forest ecosystems. Hydraulic failure and carbon starvation are two commonly proposed mechanisms for drought-induced tree die-off. Here, we show that inhibited branchlet respiration and soil-to-leaf hydraulic conductance, likely caused by cell damage, occur prior to hydraulic failure (xylem embolism) and carbon starvation (exhaustion of stored carbon in sapwood) in a drought-tolerant woody species, *Rhaphiolepis wrightiana* Maxim. The ratio of the total leaf area to the twig sap area was used as a health indicator after drought damage. Six adult trees with different levels of tree health and one dead adult tree were selected. Two individuals having the worst and second worst health among the six live trees died three months after our study was conducted. Soil-to-leaf hydraulic conductance and leaf gas exchange rates decreased linearly as tree health declined, whereas xylem cavitation and total non-structural carbon remained unchanged in the branchlets except in the dead and most unhealthy trees. Respiration rates and the number of living cells in the sapwood decreased linearly as tree health declined. This study is the first report on the importance of dehydration tolerance and respiration maintenance in living cells.

## Introduction

Global climate change has altered the annual patterns and seasonal distribution of rainfall^1^, which greatly affects forest ecosystems through drought-induced tree die-off ^2–4^. Elucidating the physiological mechanisms underlying drought-induced tree die-off is crucial for predicting future damage to global forests; however, understanding of these mechanisms remains poor^5^. Recently, two major hypotheses have been proposed to explain non-pathogenic, drought-induced tree die-off: the hydraulic failure hypothesis, which postulates that die-off is largely a result of dysfunctional water transport systems caused by embolisms in the xylem^6^; and the carbon starvation hypothesis, which suggests that die-off is caused by shortages of stored carbohydrates resulting from a decline in photosynthetic activity because of stomatal closure^5^. To date, evidence from field studies of mature trees has largely supported the hydraulic failure hypothesis over the carbon starvation hypothesis^7, 8^, whereas evidence from experimental studies of tree seedlings has supported the carbon starvation hypothesis^9^.

Drought often causes cell damage because of active oxygen species^10^ and induces xylem and phloem cell dehydration^11^. Trees cannot consume all of their stored carbon when enzymes are unable to gain access to the inner nucleus of starch granules^12^ or when carbon is sequestered in compartments from which it cannot be retrieved^13, 14^. Therefore, the failure of carbon metabolism in cells and the inability to translocate carbon within plants likely causes substantial damage to trees before the carbon reserves are exhausted. Metabolic failure decreases ATP synthesis in mitochondria^15^, and inhibited ATP synthesis drastically weakens hydraulic functions in the cell membrane as a consequence of decreased aquaporin activity^16^. The depletion of aquaporin activity leads to phloem turgor failure^17, 18^, which may negatively affect phloem transport^19^. Therefore, drought-induced tree die-off is likely related to carbon starvation and hydraulic failure as well as to metabolic failure. However, few studies have attempted to simultaneously assess the effects of all three factors, including cell damage (metabolic failure), on mature trees facing lethal drought^20^.

Here, we demonstrate that inhibited respiration related to dehydration-induced cell damage and decreased soil-to-leaf hydraulic conductance (*K*_soil-to-leaf_) occur before carbon starvation and hydraulic failure in the branchlets of drought-tolerant woody plants. To evaluate this tripartite relationship (hydraulic failure, carbon starvation and metabolic failure), we measured *K*_soil-to-leaf_, the percent loss of conductivity (PLC) in the branchlets, the non-structural carbon (NSC) and the respiration rates in the branchlets and at the middle and base of the stems, along with the leaf water potential, leaf gas exchange and the living cell ratio (the number of living cells/the number of total cells in parenchyma) of the branchlets. We selected six live adult *Rhaphiolepis wrightiana* Maxim. trees with different total leaf area/twig sap area ratios in their top-canopy shoots, which was considered an indicator of tree health (because dehydration progresses defoliation), as well as a dead adult tree. The studied tree species is commonly found in dry ridges with shallow soil on the Bonin Islands of Japan^21^, and its xylem has a relatively high tolerance to air-induced xylem embolism. This field study is the first report showing that respiratory stress is an important process that induces tree die-off under drought conditions in drought-tolerant woody plants. The evaluating of metabolic tolerance among tree species will help to increase our knowledge about future forest degradation caused by the forecasted drier climate.

## Results

The examined trees did not exhibit damage from feeding or pathogenic symptoms. Among the living 6 trees, the trees with the lowest and second lowest total leaf area/twig sap area ratios died within 3 months after this study was completed; they could not re-sprout over two years. The other trees are living over two years after this study. The total leaf area/twig sap area ratio is thus a good indicator of tree health.

**Figure 1:**
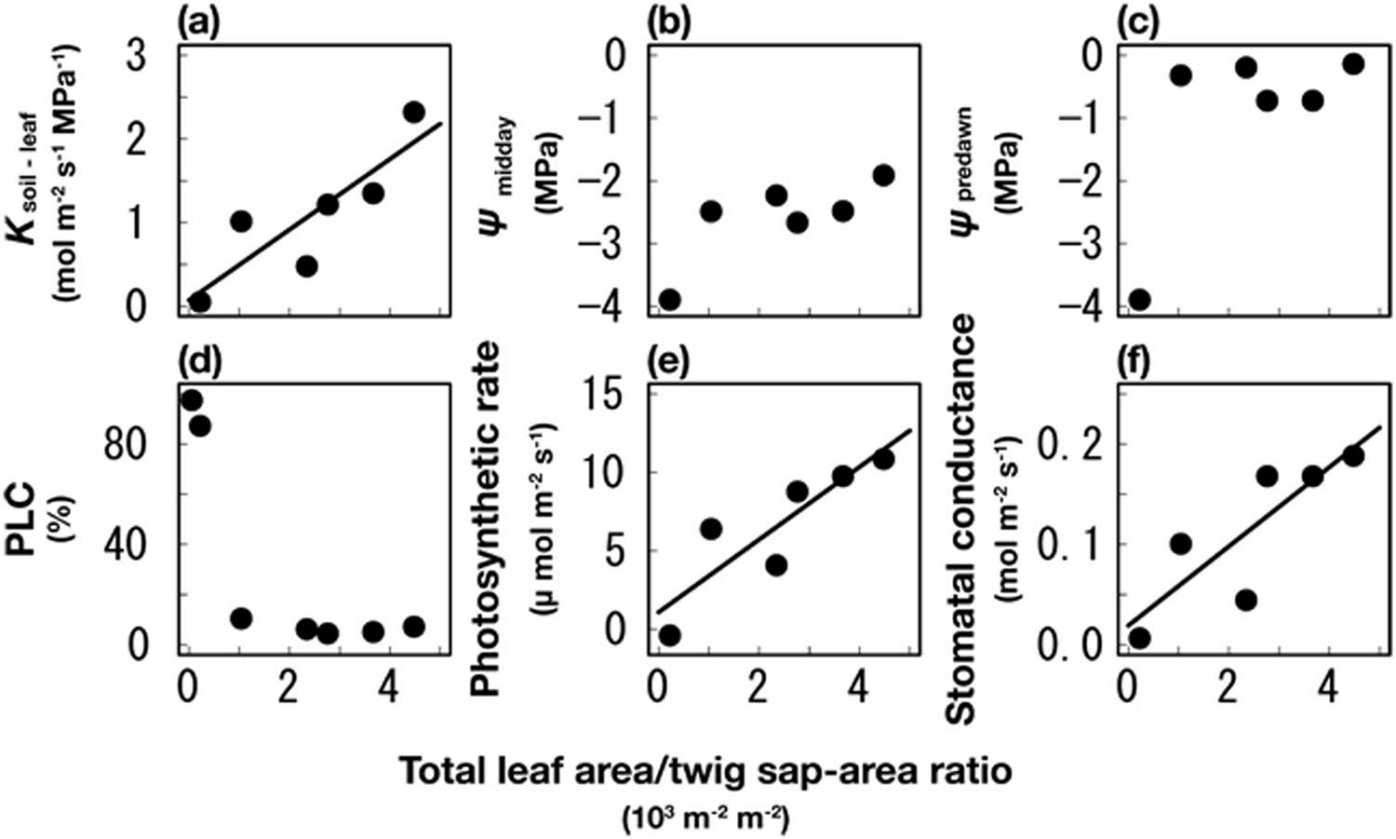
Mean hydraulic and leaf gas exchange values in individual trees with different leaf area/twig sap area ratios (degrees of tree health). (**a**) Soil-to-leaf hydraulic conductance. These values were calculated from the transpiration rates and the difference between the predawn and midday leaf water potential. (**b**) Leaf water potential at midday. (**c**) Leaf water potential at predawn. (**d**) Percentage loss of conductivity (PLC) in the branchlets (6 mm in diameter). (**e**) Near-light saturated net photosynthetic rate per unit area. (**f**) Maximum water-vapor stomatal conductance. Because the dead tree have no leaves, we could not measure leaf-rerated physiological parameters (**a**,**b**,**c**,**e** and **f**). Ordinary least-squares linear regression lines were drawn for statistically significant results (α = 0.05), and the dotted line indicates the decreasing trend of leaf water potential with declining tree health (*R* ^2^ = 0.60, *p* = 0.07). The measurement of PLC was conduced in 7 trees, including the dead tree; PLC in the dead tree was 97%.

The values of *K*_soil-to-leaf_ (Eq. 1) decreased linearly with decreases in the total leaf area/twig sap area ratio (i.e., declining tree health) (*R* ^2^ = 0.74, *p* < 0.05) (Fig. 1a) and the midday leaf water potential showed a decreasing trend (*R* ^2^ = 0.60, *p* = 0.07) with declining tree health (Fig. 1b). Because the dead tree did not have leaves, we were unable to show the parameters related to canopy leaves in this plant. The leaf water potential values at predawn remained high except in the tree with the poorest health (Fig. 1c). The PLC in the branchlets (Eq. 2) remained low (i.e., high hydraulic conductivity) except in the dead and most unhealthy trees (Fig. 1d). The results showed that the hydraulic conductance decreases between the middle stem and the root system before xylem dysfunction occurs in the branchlets. With regard to the leaf gas exchange, the photosynthetic rates and stomatal conductance decreased with declining tree health (*R* ^2^ = 0.77, *p* < 0.05 and *R* ^2^ = 0.71, *p* < 0.05, respectively) (Fig. 1e,f).

**Figure 2:**
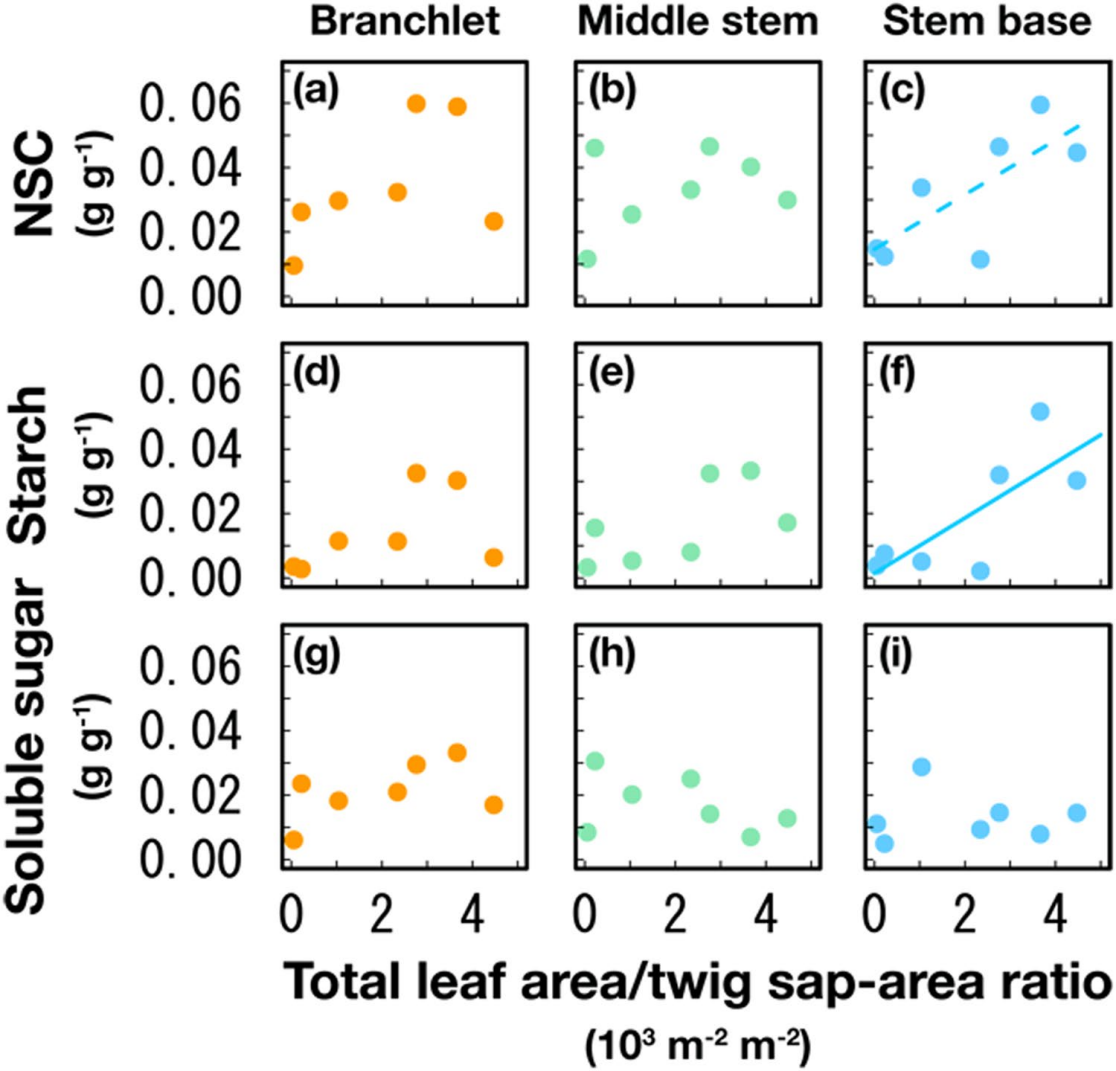
Concentrations of non-structural carbon (NSC), starch and soluble sugar in sapwood. (**a**) NSC in the branchlets (6 mm in diameter). (**b**) NSC in the middle stem (20 mm in diameter). (**c**) NSC in the stem base. (**d**) Starch in the branchlets. (**e**) Starch in the middle stem. (**f**) Starch in the stem base. (**g**) Soluble sugar in the branchlets. (**h**) Soluble sugar in the middle stem. (**i**) Soluble sugar in the stem base. The symbols indicate the branchlets (orange color), middle stem (green color) and stem base (blue color). Ordinary least-squares linear regression lines were drawn for statistically significant results (α = 0.05), and the dotted line indicates the decreasing trend of NSC in the stem base with declining tree health (*R* ^2^ = 0.56, *p* = 0.052). The measurements were conduced in 7 trees, including the dead tree.

The NSC in the branchlets and middle stem (diameter 20 mm) did not vary significantly with declining tree health (Fig. 2a,b), although a decreasing trend with declining tree health was observed in the stem base (*R* ^2^ = 0.56, *p* = 0.052) (Fig. 2c). The starch content in the stem base decreased significantly with declining tree health (*R* ^2^ = 0.58, *p* < 0.05) (Fig. 2f), although significant differences were not observed in the branchlets and middle stem (Fig. 2d,e). The soluble sugar content in all stem parts did not vary significantly with declining tree health (Fig. 2g,h,i). These facts indicate that the starch concentration at stem bases is a good indicator for tree wilting.

**Figure 3:**
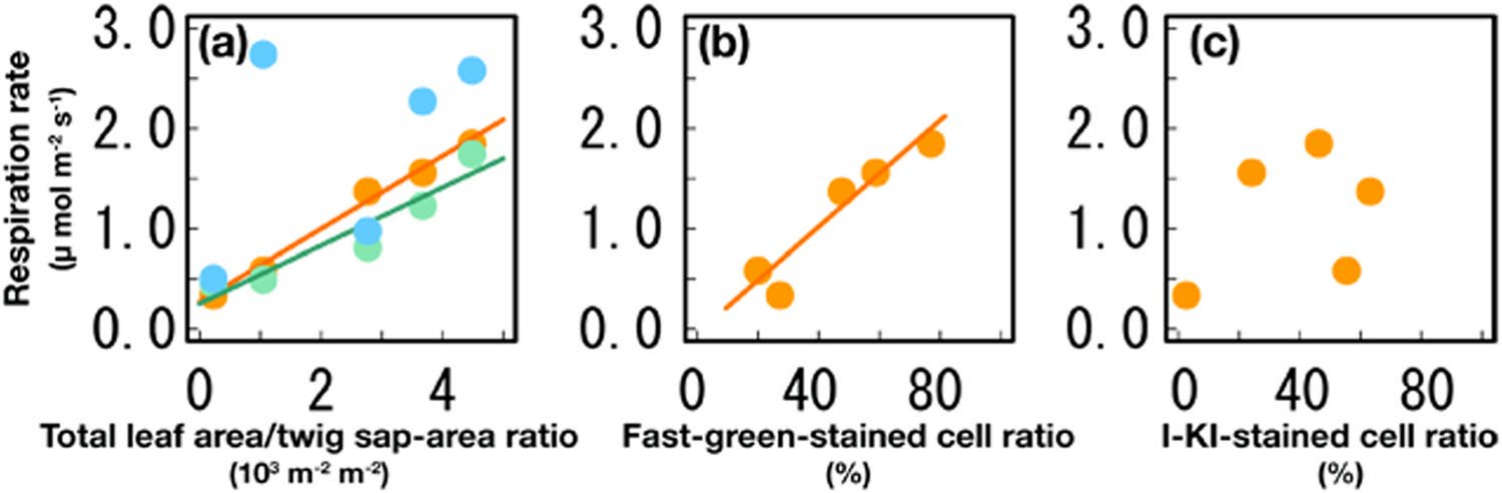
Respiration rates per unit surface area in the living trees. (**a**) Branchlets (6 mm in diameter; orange color), middle stem (20 mm in diameter; green color) and stem base (blue color). (**b**) Correlations between the ratios of the number of cells stained with fast-green to the total parenchyma cells and the respiration rates in the branchlets. (**c**) Correlations between the ratios of the number of cells with starch granules to the total parenchyma cells and the respiration rates in the branchlets. Ordinary least-squares linear regression lines were only drawn for statistically significant results (α = 0.05) (we lost data in one living tree).

Although NSC starvation was not observed in the branchlets and middle stem (Fig. 2a,b), the respiration rates decreased linearly with declining tree health (Fig. 3a; orange and green lines, respectively). However, significant variations were not observed in the stem base (Fig. 3a). Fast Green stain (061–00031, Wako Puer Chemical Industries, Tokyo, Japan) has a high affinity for the cell cytoplasm; therefore, we assessed tree health by examining the correlation between respiration rates and the number of cells stained with Fast Green. The respiration rates were positively correlated with the ratio of Fast Green-stained cells to total parenchyma cells in the sapwood of branchlets (*R* ^2^ = 0.91, *p* < 0.05) (Fig. 3b), which indicated that the decrease in respiration rates associated with decreasing tree health may have been caused by cell damage related to dehydration. However, the respiration rates were not significantly correlated with the ratio of cells with starch granules to the total parenchyma cells in the sapwood of branchlets (Fig. 3c).

## Discussion

We examined the roles of metabolic failure, carbon starvation, and hydraulic failure in the process of tree wilting and found that respiration was inhibited in the branchlets and middle stem (Fig. 3a) and *K*_soil-to-leaf_ decreased (Fig. 1a) with declining tree health prior to carbon starvation and xylem embolism in drought-tolerant woody plants. The inhibited respiration in the branchlets appears to be correlated with cell damage (Fig. 3b). Recent studies of the physiological mechanisms of tree die-off have focused on the carbon starvation and/or the hydraulic failure hypotheses. Furthermore, phloem functions (especially phloem transport) appear to play a key role in drought-induced mortality^5, 11, 19^. However, the causes and timing of phloem function failures remain unclear.

Our results show that the maintenance of active respiration is essential for tree survival during prolonged drought conditions. Stem respiration produces energy via H^+^-ATPase in the mitochondrial membrane, and this energy is used in various physiological processes that prevent dehydration, such as maintaining aquaporin-dependent hydraulic permeability^16, 22^ and phloem cell turgor^18^ and refilling embolized vessels^22–24^. The decrease in the midday leaf water potential during drought leads to cell dehydration and the collapse of cell turgor in stem cells^11^, which results in cell damage and inhibited respiration in the branchlets. The collapse of cell turgor caused by dehydration will impede carbon transport by the phloem from the leaves to roots^19, 25^, which results in decreased osmoregulatory activity^26, 27^ and fine-root productivity^25^.

The decrease in *K*_soil-to-leaf_ with tree wilting was due to reduced root function, rather than xylem embolism. Hydraulic failure in xylem dysfunction is not observed, at least in the branchlets (Fig. 1d). The decrease in *K*_soil-to-leaf_ was observed with defoliation (Fig. 1a) and defoliation has been known to increase *K*_soil-to-leaf_^28^. However, in this study, the defoliation during the process of tree wilting could not compensate for the decreased *K*_soil-to-leaf_. This fact indicates that the drastically reduced fine-root production^25^ and root hydraulic permeability^28^ are major factors for the decrease in *K*_soil-to-leaf_. The reduced root function would be associated with metabolic failure (reduced respiration) and impeded phloem transport of carbon.

The carbon stored within sapwood was not completely depleted, even in the dead tree (Fig. 2). Correlations were not observed between the respiration rates and the number of cells with starch granules in the branchlets (Fig. 3c), which may have been caused by isolated starch granules in cells. Therefore, the lack of carbon metabolism in cells and the inhibition of carbon translocation within plants are expected to cause substantial damage to trees before the complete exhaustion of their carbon reserves. Note that the remaining of NSC would not always be related to rejection of carbon starvation hypothesis, especially in high cavitation-tolerant tree species with hard wood. Therefore, comparing the ability to maintain respiration against dehydration is essential for assessing drought tolerance.

This study is the first to show that the metabolic failures associated with cell damage occur before carbon starvation and hydraulic failure in branchlets during tree wilting in anisohydric *Rhaphiolepis wrightiana* Maxim. Anisohydric trees exhibit a conspicuous decrease in daytime leaf water potential under soil desiccation because of stomatal opening, whereas isohydric trees maintain high leaf water potential under soil desiccation because of stomatal closure. In general, anisohydric trees have harder wood and exhibit higher tolerance to air-induced xylem embolisms than do isohydric trees^5, 29–32^. However, anisohydric trees display lower ratios of parenchyma cells because of their hard wood, which reduces the quantity of stored carbon within sapwood^33^. Therefore, the parenchyma cells may not be able to contribute to the plant’s physiological recovery strategies, such as osmoregulation for maintaining cell turgor^26, 27^ and refilling cavitated xylem^34^, following rainfall after prolonged drought and irreversible damage may occur because of severe dehydration^35^.

## Methods

The study site was located on the Ogasawara (Bonin) Islands of Japan (27°05′N, 142°13′E), which are in the northern Pacific Ocean. The islands are authorized as a World Natural Heritage site since 2011, because of the unique ecosystems. The study was conducted in the summers of 2014 and 2015. The mean air temperature was 23.3 °C, and the mean annual precipitation was 1,253 mm for the period from 2006 to 2015 (these data were obtained by the Japan Meteorological Agency). Climatically, the study year was normal but with a relatively wet summer.

We selected six adult *Rhaphiolepis wrightiana* Maxim. trees with different total leaf area/twig sap area ratios (i.e., different defoliation levels) in the top-canopy shoots as well as a dead tree to compare the physiological traits related to carbon, hydraulic and respiratory stress among individual trees. Before measuring, we confirmed that the examined trees did not exhibit damage from feeding or pathogenic symptoms. The trees with the worst and second worst health among the living trees died within 3 months after this study. This species mainly grows on dry ridges with shallow soil (mean soil depth of 0.24 m), and they present relatively high cavitation tolerance in the xylem (mean *P*_50_ = −6.4 MPa) (*P*_50_ = the daytime xylem water potential corresponding to 50% loss of conductivity). The leaf gas exchange was measured with an LI-6400 system (LI-COR, Lincoln, NE) from the morning to midday (10:00–12:00). The leaf water potential was measured before dawn (02:00–04:00) and again at around midday (immediately after the measurement of leaf gas exchange) with a pressure chamber (Model 1505-D-EXP, PMS, Corvallis, OR). Soil-to-leaf hydraulic conductance (*K*_soil-to-leaf_; mol m^−2^ s^−1^ MPa^−1^) was calculated, as follows;

1 $${K}_{\mathrm{soil} \mbox{-} \mathrm{to} \mbox{-} \mathrm{leaf}}=E/({\psi }_{{\rm{pre}}}-{\psi }_{{\rm{mid}}})$$

where *E* is leaf-area based transpiration rate (mol m^−2^ s^−1^) and *ψ*_pre_ and *ψ*_mid_ are predawn and midday leaf water potential, respectively; assuming that *ψ*_pre_ is equilibrium to soil water potential in root systems. The leaf gas exchange was measured in 6 living trees.

The stem respiration rates in the stem base (diameter of 60–120 mm) and the middle stem (diameter of 20 mm) were measured in the study field at night (00:00–04:00) when the partial pressure and temperature were not relatively fluctuated and when the sap flow in the stem has nearly ceased^36–41^. The respiration rates in the branchlets (diameter of 6 mm) were measured in our laboratory immediately after sampling (room temperature of 25 °C) in 5 living trees, except for the dead tree and one living tree (because of data lost). The respiration rates were calculated from changes in the CO_2_ concentrations in closed chambers, and the CO_2_ concentrations were measured with a thin-film capacitance sensor (Model GM70, Vaisala, Helsinki, Finland). To adjust the partial pressure of the CO_2_ in the closed chambers, we monitored the temperature of the closed chamber in all respiration measurements.

We measured PLC (percent loss of conductivity) in branchlets. We measured the maximum vessel length of branch (40 ± 30 mm, mean ± 1 SD), using air-injection methods^42^. For PLC measurements, 4 branches of 0.3–0.5 m in length were harvested from an individual tree before dawn, and recut under water in the field. We immediately removed the collected shoots to our laboratory, and the shoots were reserved under dim light during 4 hours to remove negative pressure in xylem. After that, we recut the twigs under water again to collect branch segments for measuring twig hydraulic conductivity. We measured the initial hydraulic conductivity (*K*_i_) in 2 or 4 collected branchlets with a length of approximately 50 mm and a diameter of approximately 6 mm. The downside end of branch segments was pressured by 0.2 µm-filtered 20 mM KCl solution with gravimetric 5-kPa hydraulic pressure (50 cm high) via a tubing system; the other end was connected to an electric balance via a tubing system^43^. All of the hydraulic measurements were performed under a temperature of 25 °C. The PLC was calculated as follows:

2 $${\rm{PLC}}=1-({K}_{{\rm{i}}}/{K}_{{\rm{\max }}})\times 100,$$

where *K*_i_ is the initial hydraulic conductivity and *K*_max_ is the maximum hydraulic conductivity. The values of *K*_max_ were measured in the branch segments, after adding 0.1 MPa hydraulic pressure (KCl solution) during 15 min from the downside end to remove air-induced embolism.

The NSC contents (starch and soluble sugars) of the xylem sapwood were measured in the branchlets, the middle stem and the stem base. The sapwood (without the bark and pith) was ground to a fine powder and then extracted in 80% ethanol (v/v). The supernatant was extracted by centrifuging and used to quantify the soluble sugar contents via the phenol–sulfuric acid method. The starch in the remaining pellets was depolymerized to glucose by the addition of KOH, acetic acid and amyloglucosidase buffer. After quantifying the glucose extracted via the mutarotase–glucose oxidase method (Glucose C-II test, Wako, Tokyo, Japan), the starch contents within the xylem sapwood were measured.

**Figure 4:**
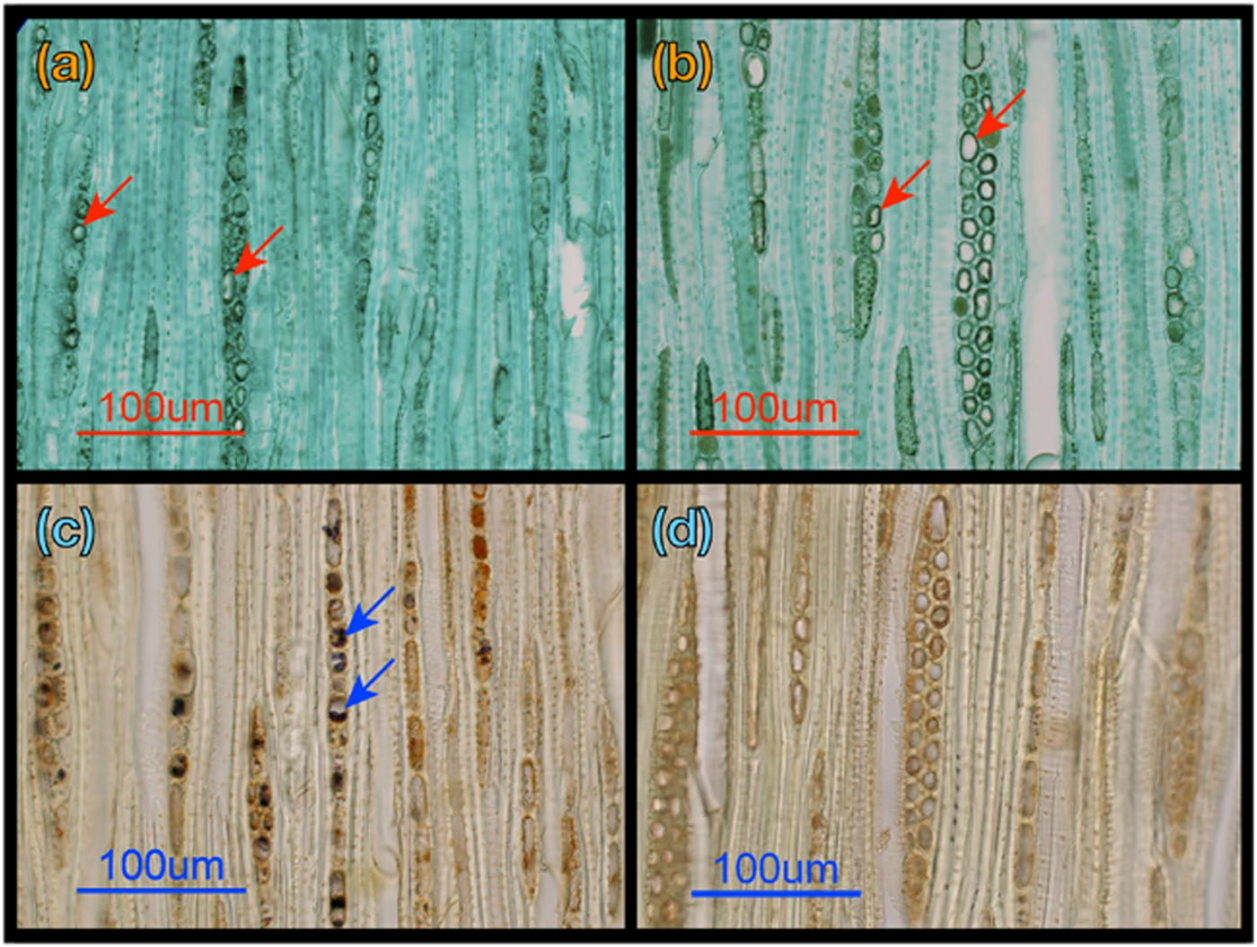
Sections tangential to the tree ring stained with Fast Green, which has a high cellar cytoplasm affinity, and with iodine–potassium iodine (I–KI) solution, which stains starch granules in the sapwood of branchlets. (**a**) Section from the healthiest tree stained with Fast Green. (**b**) Section from the least healthy living tree stained with Fast Green. (**c**) Section from the healthiest tree stained with I–KI solution. (**d**) Section from the least healthy living tree stained with I–KI solution. In panels (a) and (b) red arrows show the cells counted as damaged cells. In panel (c) blue arrows show starch granules in the cells. The length of the scale bars is 100 µm.

The branchlets were fixed in FAA (10% formaldehyde, 5% acetic acid and 50% ethanol) immediately after sampling. To detect cell damage and starch in the cells, tangential sections (15 µm in thickness) of the branchlets were cut with a sliding microtome (HM 400 R, Micron, Walldorf, Germany). The successive sections tangential to the tree ring were stained with Fast Green, which has a high cellar cytoplasm affinity, and with iodine–potassium iodine (I–KI) solution, which stains the starch granules. Figure 4 shows sections tangential to the growth ring in the sapwood of the branchlets stained with Fast Green and the I–KI solution in the healthiest and least healthy individual trees among the living trees. We counted the stained and non-stained parenchyma cells in 2–4 branchlets of each individual (the average number of counted cells was 798 in each branchlet). The ratio of stained cells to the total parenchyma cells was calculated in the stained sections, and the ratio of cells with starch granules to total the parenchyma cells was counted in the I–KI-stained sections. In the Fast Green stain, the cells in which the cytoplasm was not fully stained were counted as damaged cells (red arrows in Fig. 4a,b).

The comparisons between the parameters and the sum of the leaf area per unit sap area and between the respiration rates in the branchlets and the ratios of stained cells were analyzed by ordinary least-squares linear regressions (α = 0.05). These statistical analyses were conducted with the glm R function (version 2.151) (R Development Core Team).
